# Valine biosynthesis in *Saccharomyces cerevisiae *is regulated by the mitochondrial branched-chain amino acid aminotransferase Bat1

**DOI:** 10.15698/mic2018.06.637

**Published:** 2018-03-21

**Authors:** Natthaporn Takpho, Daisuke Watanabe, Hiroshi Takagi

**Affiliations:** 1 Graduate School of Biological Sciences, Nara Institute of Science and Technology, 8916-5 Takayama, Ikoma, Nara 630-0192, Japan.

**Keywords:** yeast Saccharomyces cerevisiae, valine, mitochondria, Bat1, branched-chain amino acid aminotransferase

## Abstract

In the yeast *Saccharomyces cerevisiae*, the branched-chain amino acid aminotransferases (BCATs) Bat1 and Bat2 catalyze the conversion of α-ketoisovalerate, α-keto-β-methylvalerate, and α-ketoisokaproate and into valine, isoleucine, and leucine, respectively, as the final step of branched-chain amino acid biosynthesis. Bat1 and Bat2 are homologous proteins that share 77% identity, but Bat1 localizes in the mitochondria and Bat2 in the cytosol. Based on our preliminary finding that only disruption of the *BAT1* gene led to slow-growth phenotype, we hypothesized that Bat1 and Bat2 play distinct roles in valine biosynthesis and the regulation of cell growth. In this study, we found that intracellular valine content was dramatically decreased in Δ*bat1* cells, whereas Δ*bat2* cells exhibited no changes in the valine level. To further examine the distinct roles of Bat1 and Bat2, we constructed two artificial genes encoding the mitochondrial-targeting signal (MTS)-deleted Bat1 (Bat1-MTS) and the MTS of Bat1-fused Bat2 (Bat2+MTS). Interestingly, Bat2+MTS was relocalized into the mitochondria, because Bat2 localization was changed to the mitochondria by addition of MTS, and could partially restore the valine content and growth in Δ*bat1*Δ*bat2* cells. These results suggest that the mitochondria are the major site of valine biosynthesis, and mitochondrial BCAT is important for valine biosynthesis in *S. cerevisiae*.

## INTRODUCTION

The metabolism of branched-chain amino acids (BCAAs), such as valine, isoleucine, and leucine, has been intensively studied in mammals as its deficiency is linked to several human diseases 1. Metabolism of BCAAs is also associated with protein, glucose, and energy metabolism 2. It is generally known that BCAAs cannot be synthesized in mammals, and thus only the catabolic pathway has been well characterized. Catabolism of BCAAs in mammals is mostly carried out in the mitochondria and produces acetyl-CoA and succinyl-CoA as the end products. BCAA aminotransferases (BCATs) catalyze the first step of BCAAs catabolism, in which the transamination of valine, leucine, and isoleucine to α-ketoisovalerate (KIV), α-ketoisocaproate (KIC), and α-keto-β-methylvalerate (KMV), respectively, occurs. In eukaryotic organisms, there are two isoforms of BCATs, mitochondrial BCAT (mBCAT) and cytosolic BCAT (cBCAT), which correspond to Bat1 and Bat2 in the yeast *Saccharomyces cerevisiae*, respectively. In general, mBCAT is primarily involved in the initiation of BCAAs catabolism. On the other hand, cBCAT is likely to be associated with brain function by playing an important role in the nitrogen shuttle of glutamate, a neurotransmitter 3. In addition to BCAT, most catabolic reactions involve the mitochondrial enzyme, branched-chain α-ketodehydrogenase (BCKD). It has been reported that the human BCATs function as heterodimers and homodimers 4, but only the homodimer structure is in an active form in *S. cerevisiae* 5. The distribution of theses isoforms depends on the requirement of nitrogen metabolism in each cellular component 6.

Unlike in higher eukaryotes, the *S. cerevisiae* BCATs, Bat1 and Bat2, could function in both anabolic and catabolic pathways as the final step in the biosynthesis and the first step in the degradation of BCAAs. Although Bat1 and Bat2 are homologous proteins with 77% primary sequence identity, their localizations are clearly different. Bat1, which has a mitochondrial-targeting signal (MTS), is localized in the mitochondria, whereas Bat2, which lacks the MTS, is present in the cytosol. To investigate the different roles of Bat1 and Bat2, we previously constructed yeast strains with a single gene disruption of *BAT1 *or *BAT2 *and observed that only Δ*bat1* cells showed the slow-growth phenotype. This result suggested that Bat1 and Bat2 play distinct roles in BCAAs biosynthesis. Accordingly we here investigated the specific roles of Bat1 and Bat2 in the valine level and growth of yeast cells.

## RESULTS 

**Figure 1:**
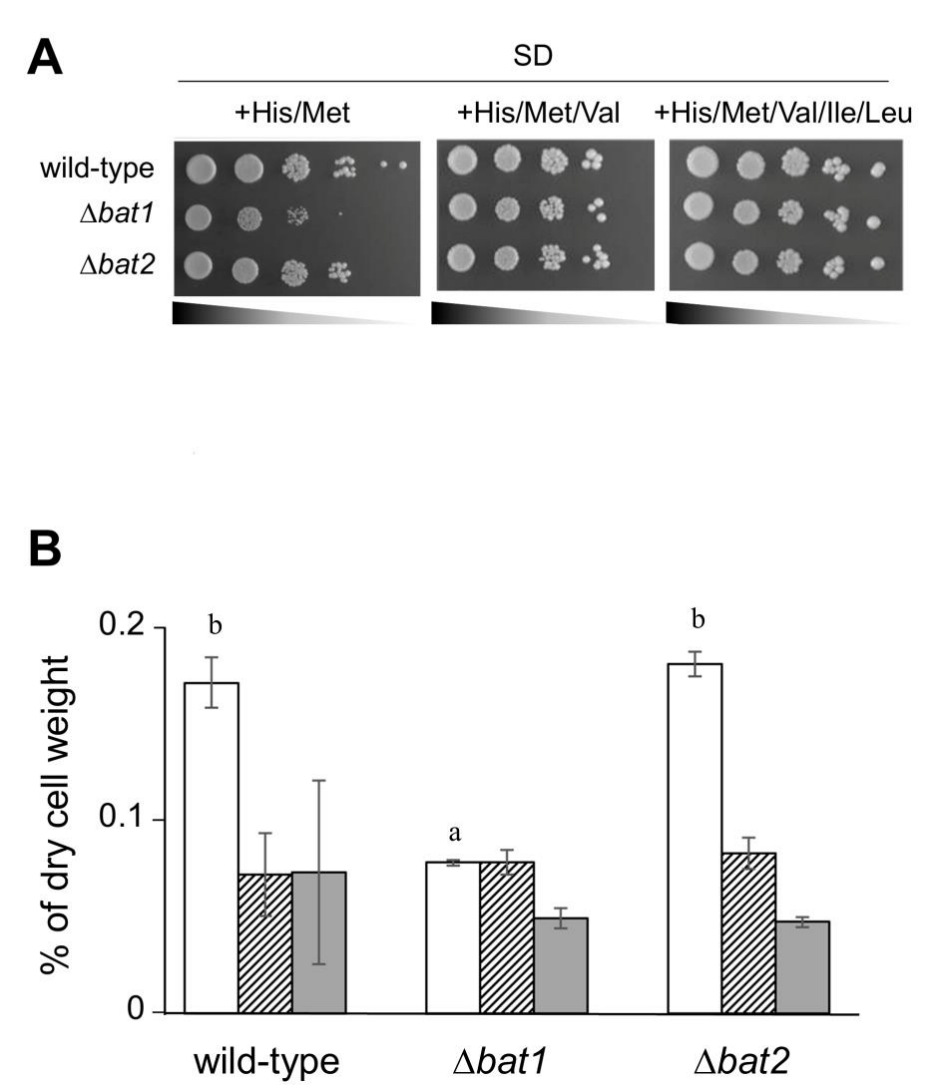
FIGURE 1: Effects of Bat1 and Bat2 on the growth and BCAAs contents of yeast cells. **(A)** Growth phenotypes of *S. cerevisiae* wild-type (BY4741), ∆*bat1*, and ∆*bat2* cells harboring pRS415/pRS416 cultured on SD+His/Met, SD+His/Met/Val or SD+His/Met/Val//Ile/Leu medium. **(B)** Intracellular BCAAs contents of *S. cerevisiae* wild-type (BY4741), ∆*bat1*, and ∆*bat2 *cells harboring pRS415/pRS416 cultured in SD+His/Met medium. White, striped, and dark gray bars represent intracellular valine, leucine, and isoleucine contents, respectively. The values are the means and standard deviations of three independent experiments. Statistically significant differences were determined by Student’s *t* test (^a,b^p <0.01).

**Figure 2:**
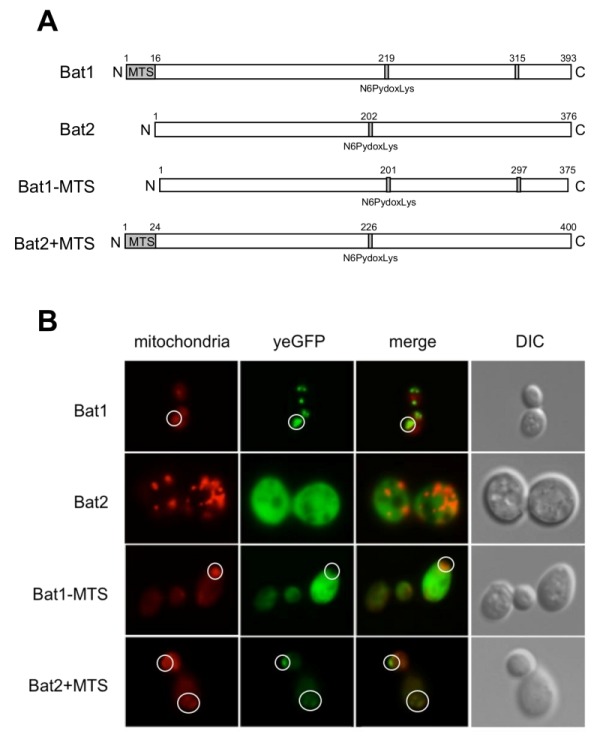
FIGURE 2: Subcellular localization of Bat1 and Bat2. **(A)** Schematic structure of Bat1, Bat2, Bat1-MTS, and Bat2+MTS based on UniProt 10. **(B)** Subcellular localization of Bat1, Bat2, Bat1-MTS, and Bat2+MTS by fluorescent microscopy. *S. cerevisiae* wild-type (BY4741) cells harboring pRS416-Bat1-yeGFP/pRS415 (Bat1), pRS416-Bat1-MTS-yeGFP/pRS415 (Mat1-MTS), pRS415-Bat2-yeGFP/pRS416 (Bat2), and pRS415-Bat2+MTS-yeGFP/pRS416 (bat2+MTS) were grown in SD+His/Met medium at 30ºC for 15 h. MitoTracker signals (mitochondria), GFP signals (yeGFP), merged signals (merged), and differential interference contrast images (DIC) are shown. White circles show the mitochondria in Bat1, Bat1-MTS and Bat2+MTS.

Our preliminary experiment revealed that ∆*bat1* cells exhibited slower growth than wild-type and ∆*bat2* cells under amino acid-starved conditions and the addition of valine or BCAAs could recover the slow-growth of ∆*bat1* cells (Figure 1A), suggesting that Bat1, not Bat2, plays a predominant role in BCAAs synthesis. In fact, the level of intracellular valine was significantly lower in ∆*bat1* cells compared with wild-type and ∆*bat2* cells (Figure 1B). Intriguingly, the levels of leucine and isoleucine were unaffected by gene deletion of either *BAT1* or *BAT2*, suggesting that the mitochondrial synthesis of valine catalyzed by Bat1 is more important than its cytosolic synthesis through the reaction by Bat2. In order to address this hypothesis, we constructed a cytosolic Bat1 (without MTS) and a mitochondrial Bat2 (with MTS). The predicted MTS was removed from the amino-terminus of Bat1 (18 amino acids), which we designated Bat1-MTS, while 24 amino-terminal residues of Bat1 were attached to the amino-terminus of Bat2, which we named Bat2+MTS (Figure 2A). The localization of these engineered proteins was determined by tagging the GFP at the carboxyl terminus of each protein and observed under fluorescent microscopic. The results clearly showed that there was no mitochondrial localization in Bat1-MTS, whereas Bat2+MTS was relocalized into the mitochondria (Figure 2B), indicating that these modifications altered the native localization of Bat1 and Bat2.

**Figure 3:**
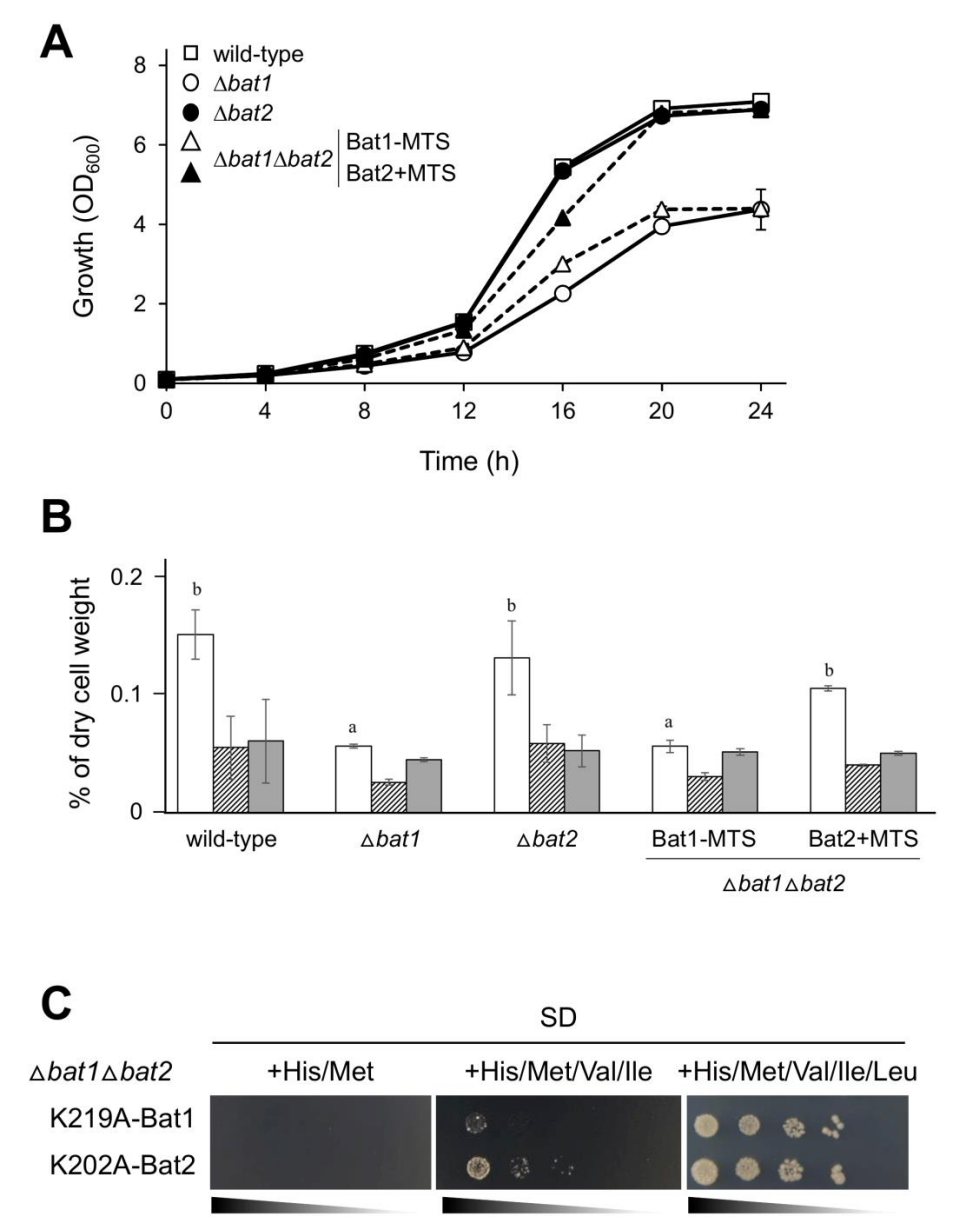
FIGURE 3: Effects of mitochondrial BCAT on the growth and BCAAs contents of yeast cells. **(A)** Growth phenotype of *S. cerevisiae* wild-type (BY4741) (open square), ∆*bat1* (open circle), ∆*bat2* (closed circle), and ∆*bat1*∆*bat2* cells harboring pRS416-Bat1-MTS/pRS415 (open triangle) or pRS415-Bat2+MTS/pRS416 (closed triangle) in SD+His/ Met liquid medium. The values are the means and standard deviations of three independent experiments. **(B)** Intracellular BCAAs contents in *S. cerevisiae* wild-type (BY4741), ∆*bat1*, ∆*bat2*, and ∆*bat1*∆*bat2* cells harborin pRS416-Bat1-MTS/pRS415 or pRS415-Bat2+MTS/pRS416 cultured in SD+His/Met medium. White, striped, and dark gray bars represent intracellular valine, leucine, and isoleucine contents, respectively. Data shown are the average and the standard deviation of three independent experiments. The values are the means and standard deviations of three independent experiments. Statistically significant differences were determined by Student’s *t* test (^a,b^*p *<0.05). **(C)** Growth phenotype of *S. cerevisiae* ∆*bat1*∆*bat2* cells harboring pRS416-K219A-Bat1 and pRS415-K202A-Bat2 cultured on SD+His/Met, SD+His/Met/Val/Ile, and SD+His/Met/Val/Ile/Leu medium, respectively

Next, we confirmed that Bat1-MTS and Bat2+MTS functioned properly in ∆*bat1*∆*bat2* cells. As shown in Figure 3A, ∆*bat1* cells clearly exhibited a slow-growth in liquid minimal medium as compared with wild-type and ∆*bat2* cells. In addition, ∆*bat1*∆*bat2* cells harboring Bat1-MTS grew similarly to ∆*bat1* cells. In contrast, the expression of Bat2+MTS fully restored the growth of ∆*bat1*∆*bat2* cells to the same level as in wild-type and ∆*bat2* cells. These results suggested that both Bat1-MTS and Bat2+MTS were basically functional in BCAAs synthesis. More importantly, Bat1 and Bat2+MTS have a predominant role in cell growth via valine biosynthesis as the mitochondrial BCATs, while Bat2 and Bat1-MTS have a minor role as the cytosolic BCATs. To determine whether the growth restoration in ∆*bat1*∆*bat2* cells expressing Bat2+MTS was associated with the intracellular valine content, we measured the cellular levels of BCAAs (Figure 3B). The results clearly indicated that the valine content in ∆*bat1*∆*bat2* cells harboring Bat1-MTS was similar to that in ∆*bat1* cells. On the other hand, the valine level in ∆*bat1*∆*bat2* cells harboring Bat2+MTS was almost identical to the valine levels in wild-type and ∆*bat2* cells. Taken together, these findings suggest that mitochondrial BCAT is required for the effective synthesis of valine, since the BCAT activity of Bat2+MTS can fulfill the valine biosynthesis by significantly increasing the intracellular valine content to a level comparable to that in wild-type and ∆*bat2* cells, leading to growth restoration.

It should be noted that expression of inactive BCATs, Lys219Ala-Bat1 and Lys202Ala-Bat2 7, did not recover the growth of ∆*bat1*∆*bat2* cells in BCAA-depleted or leucine-depleted medium (Figure 3C). Also, it was notable that the combination of exogenous valine and isoleucine did not support the growth of ∆*bat1*∆*bat2* cells harboring Lys219Ala-Bat1 and Lys202Ala-Bat2. In this experiment, we did not add leucine to the SD medium for maintenance of the plasmid stability. When all BCAAs (valine, leucine, and isoleucine) were added, ∆*bat1*∆*bat2* cells harboring both inactive Bat1 and Bat2 were grown on SD medium (Figure 3C). The results showed that addition of leucine could recover the growth of ∆*bat1*∆*bat2* cells, suggesting that leucine has another specific role in the cell. However, the growth of these mutants could not be fully recovered as it could in the wild-type and ∆*bat2* cells on SD medium without BCAAs, suggesting that intracellular BCAAs are required to maintain the normal cell growth.

## DISCUSSION

BCAT in *S. cerevisiae* consists of two isoforms, Bat1 and Bat2. These two proteins are homologous to ECA39 and ECA40 in mammals, which are located in mitochondria and cytosol, respectively 8. Our study revealed that deficiency in Bat1, not in Bat2, led to the slow-growth phenotype in *S. cerevisiae*. This phenomenon caused a dramatic decrease in intracellular valine content. A previous study reported that Bat1 and Bat2 have distinct functions - namely, Bat1 activity is required mainly for BCAAs biosynthesis, while Bat2 appears to play roles in the catabolic pathway 9. In this study, the putative cytosolic Bat1 (Bat1-MTS) and mitochondrial Bat2 (Bat2+MTS) were constructed based on the protein database (UniProt) 10. However, the deletion of 24 and 30 amino acid residues from the amino terminus of Bat1 impaired the BCAT activity (data not shown), while the removal of only 18 amino acids resulted in relocalization of Bat1 from the mitochondria to cytosol without perturbing the BCAT activity. These results suggest that these amino acid residues are responsible for the mitochondrial localization.

Our study also showed that Bat1-MTS could fulfill the roles of Bat2 and that the function of Bat1 was complemented by Bat2+MTS. This suggests that, when either Bat1 or Bat2 is localized into mitochondria, it can enable the valine biosynthesis and restore the cell growth. This result, in turn, indicates that mitochondrial BCAT is important for valine biosynthesis and regulates cell growth through the valine homeostasis. Meanwhile, neither *∆bat1∆bat2* cells harboring inactive Bat1 nor Bat2 could grow on minimal medium in the absence of BCAAs. This result strongly supports the idea that BCAT activity is important for cell growth. On the other hand, the addition of valine and isoleucine to the minimal medium did not restore growth of *∆bat1∆bat2* cells to a level the comparable to that of wild-type or single *∆bat1* and *∆bat2* cells, suggesting that BCATs associate with other cellular metabolic pathways, such as the TORC1 signaling pathway. The TORC1 pathway plays role in the regulation of yeast cell growth through the phosphorylation of three major effectors branches, and BCAAs are involved in the regulation of TORC1 7,11. Moreover, the absence of Bat1 and Bat2 activity has also been shown to affect homeostasis of metabolites in the central metabolic pathway, such as glutamate and glutamine 12. Such results are in good agreement with our present finding that addition of valine and isoleucine could not recover the growth of *∆bat1∆bat2* cells harboring inactive Bat1 and Bat2 due to the lack of leucine and BCAT activity. In this context, it is worth noting that intracellular BCAAs are products of a biosynthetic pathway, and thus mainly produced in mitochondria, whereas exogenous BCAAs clearly enter the catabolic pathway in cytosol. Therefore, the transportation of BCAAs including their α-ketoacids from mitochondria to cytosol or cytosol to mitochondria should affect the biosynthesis or catabolism of BCAAs as well. However, neither the levels of mitochondrial BCAAs nor the levels of cytosolic BCAAs were quantified in this study, since no effective methods were available for these measurements. Moreover, the evidence in regard to the transportation of BCAAs, including other amino acids, either into or out of the mitochondria, remains unclear; therefore, the regulatory mechanisms of BCAAs metabolism under biosynthetic and catabolic conditions must be further studied.

Based on our present findings, we propose that mitochondrial BCAT is required for effective valine biosynthesis and plays a role in growth regulation, and we conclude that mitochondria are the main source of intracellular valine. It is notable that mitochondrial BCAT plays a major role in valine biosynthesis by converting mitochondrial KIV into valine, whereas cytosolic KIV is partially metabolized by the Ehrlich pathway, as previously reported 13. Taken together, our results in yeast suggest that mitochondrial BCAT is important for valine biosynthesis based on the following facts. (1) Cytosolic KIV is partially converted into fusel acids and isobuthanol via the Ehrlich pathway, and thus the major valine source is produced in mitochondria. (2) Only Bat1 acts as a mitochondrial BCAT responsible for valine biosynthesis in mitochondria, and thus Bat2 with MTS can replace mitochondrial BCAT activity at almost the same rate as Bat1 and maintain normal valine biosynthesis. (3) The confined environment of mitochondria promotes the BCAT enzymatic reactions, thereby accelerating the conversion of KIV into valine. (4) Since KIV is only produced in mitochondria, it can be directly converted into valine without any transport limitation.

The *bat1* mutations affect valine but not leucine and isoleucine biosynthesis. Bat1 probably associates with substrate specificity of each ketoacid, KIV, KIC, and KMV for valine, leucine, and isoleucine, respectively. The previous study 9 showed that the highest transaminase activity was observed when KIV was used as a substrate and the lowest was observed by KMV, suggesting that each BCAA intermediate is differently interact with BCATs enzyme. It cannot be truly said that all the transaminase activity come from Bat1 or Bat2 since there are other unknown transaminases in the cell. Moreover, KIC is produced in cytosol and partially transported back to mitochondria where the last step is proceeded by Bat1 and Bat2, suggesting that mitochondria is not the major compartment for leucine biosynthesis. In addition, the (-isopropylmalate synthase Leu4/Leu9, not Bat1, plays a major role in conversion of KIV to (-isopropylmalate, which is subsequently converted into KIC in cytosol, suggesting that lacking of Bat1 has the less effect on leucine biosynthesis.

## MATERIALS AND METHODS

### Strains

Yeast strains used in this study were the *S. cerevisiae* strains with a BY4741 (*MAT***a ***his3*Δ*1 leu2*Δ*0 met15*Δ*0 ura3*Δ*0*) background (provided by Open Biosystems or National Bio-Resource Project (NBRP) of the MEXT, Japan) (Table 1). *S. cerevisiae* ∆*bat1*, ∆*bat2*, and ∆*bat1*∆*bat2* strains were constructed by PCR-mediated gene disruption from strain BY4741. PCR toolbox 14 was employed in this study using pFA6a-*kanMX6 *(Addgene) and pFA6a-*hphNT1* (Euroscarf) as a template for amplification of the *kanMX6* and *hphNT1* cassettes, respectively, using gene-specific oligonucleotide primers. Target gene was replaced by the *kanMX6* or *hphNT1* cassette using high-efficiency transformation method based on homologous recombination 15. Gene disruptants were selected on YPD medium containing 150 µg/mL geneticin or 50 µg/mL hygromycin B. Selected yeast strains were confirmed by colony PCR using 500 bp up- and down-stream primers of the target genes. An *Escherichia coli* DH5α strain (*F^-^, (80dlacZ*(*M15, *(*(lacZYA-argF) U169, deoR, recA1, endA1, hdR17 (r_k_^-^, m_k_^+^), phoA, supE44, (^-^, thi-1, gyrA96, relA1*) was used for plasmid propagation and the bacterial transformation was carried out by the high efficiency transformation method 16.

**Table 1:** Yeast strains and plasmids used in this study.

Strain or plasmid	Genotype
BY4741	*MAT***a*** his3∆1 leu2∆0 met15∆0 ura3∆0*
*∆bat1*	BY4741 Δ*bat1::kanMX6*
*∆bat2*	BY4741 Δ*bat2::kanMX6*
*∆bat1∆bat2*	BY4741 Δ*bat1::kanMX6* Δ*bat2::hphNT1*
pRS416	*CEN6 URA3*
pRS416-Bat1	*BAT1* in pRS416
pRS416-Bat1-yeGFP	*BAT1-yeGFP* in pRS416
pRS416-Bat1-MTS	*BAT1ΔN18* in pRS416
pRS416-Bat1-MTS-yeGFP	*BAT1ΔN18-yeGFP* in pRS416
pRS416-K219A-Bat1	*bat1*^K219A^ in pRS416
pRS415	*CEN6 LEU2*
pRS415-CgHIS3MET15	*Candida glabrata HIS3 MET15* in pRS415
pRS415-Bat2	*BAT2* in pRS415
pRS415-Bat2-yeGFP	*BAT2-yeGFP* in pRS415
pRS415-Bat2+MTS	*BAT2+MTS* in pRS415
pRS415-Bat2+MTS-yeGFP	*BAT2+MTS-yeGFP* in pRS415
pRS415-K202A-Bat2	*bat2*^K202A^ in pRS415
	

### Plasmids

Yeast centromere plasmids (YCp) were employed for the construction of the *E. coli*-*S. cerevisiae* shuttle vectors in this study (Table 1). Plasmids pRS415 and pRS416 contain the *S. cerevisiae* *LEU2* and *URA3* gene as selection markers, respectively. Plasmids pRS416 and pRS415-CgHIS3MET15 (constructed by S. Morigasaki) were co-transformed into yeast cells to complement the auxotrophy of strain BY4741. The genes encoding Bat1, Bat1-MTS, Bat2, and Bat2+MTS were inserted to pRS416 (Bat1 series) and pRS415 (Bat2 series) at the *Sma*I and *Not*I recognition sites to construct pRS416-Bat1, pRS416-Bat1-MTS, pRS415-Bat2, and pRS415-Bat2+MTS. The genes encoding Bat1, Bat1-MTS, Bat2, and Bat2+MTS were also fused yeGFP at the carboxyl terminus using pYM25 (Euroscarf) and inserted to pRS416 (Bat1 series) and pRS415 (Bat2 series) at the *Sma*I and *Not*I recognition sites to construct pRS416-Bat1-yeGFP, pRS416-Bat1-MTS-yeGFP, pRS415-Bat2-yeGFP, and pRS415-Bat2+MTS-yeGFP. Site-direct mutagenesis was used to constructs pRS416-K219A-Bat1 and pRS415-K202A-Bat2 using mutagenic primers. PCR products were then digested with *Dpn*I before introduction into *E. coli* DH5α cells 16. DNA sequence was confirmed by DNA sequencing using BigDye® Terminator v3.1 Cycle Sequencing Kit (Applied Biosystems). Transformants of *E. coli *DH5α cells were selected based on blue-white colony screening. Plasmids were subsequently transformed to wild-type cells for observation of protein localization or to *∆bat1∆bat2* cells for phenotypic study.

### Culture Media

The wild-type strain was grown in a nutrient YPD medium (10 g/L yeast extract, 20 g/L peptone, and 20 g/L glucose) at 30°C. A synthetic dextrose minimal (SD) medium (1.7 g/L yeast nitrogen base without amino acid and ammonium sulfate (Difco Laboratotries), 5 g/L ammonium sulfate, and 20 g/L glucose) was used for *S. cerevisiae* BY4741 cells harboring pRS416 and pRS415-CgHIS3MET15, and SD medium supplemented with histidine (20 mg/L) and methionine (20 mg/L) (SD+His/Met) was used for cells harboring pRS416 and pRS415 to maintain the transformant status. Solid medium was prepared by addition of 2% agar. Valine, leucine, and isoleucine were individually added to SD+His/Met medium as 150 mg/L, 100 mg/L, and 30 mg/L, respectively (SD+His/Met/Val/Ile/Leu). *E. coli* DH5α cells were grown at 37°C in Luria-Bertani (LB) complete medium, which contains 5 g/L yeast extract, 10 g/L tryptone and 10 g/L NaCl. SOB (20 g/L tryptone, 5 g/L yeast extract, 10 mM NaCl, 2.5 mM KCl, 10 mM MgSO_4_, and 10 mM MgCl_2_) and SOC media (SOB supplemented with 20 mM glucose) were used for competent cells preparation and transformation 16. Ampicillin (100 µg/mL) was added to the LB medium to maintain the transformant status.

### Growth Test

Fresh yeast cells were cultured in SD liquid medium for 16 h at 30°C. After harvesting and washing the cells with distilled water three times, cells corresponding to an OD_600_ of 1.0 and serial dilution of 10^-1^ to 10^-3^ were prepared and 2.5 µL of each dilution was spotted on SD agar plates. Plates were incubated at 30°C for 3 days. Growth profile study in liquid medium was carried out by culture yeast cells in SD liquid medium at the initial concentration as OD_600_ of 0.1. Cells were taken for OD_600_ measurement at 4 h interval until the culture reached the stationary phase. The data was analyzed by plotting a graph between time point and absorbance at 600 nm.

### Measurements of Intracellular Amino Acid Content

*S. cerevisiae* cells were pre-cultured in 5 mL of SD medium for 16 h at 30°C and transferred to 25 mL of SD medium for cultivation at 30°C with shaking at 200 rpm. Yeast cells corresponding to an OD_600_ of 10 were collected, washed twice with distilled water and suspended in 500 µL of distilled water. Intracellular amino acids in cell suspension were extracted by boiling for 15 min at 100°C. Cell debris was centrifuged at 13,000 x *g* for 1 min and subsequently omitted by filtration using 0.2 µm syringe filter (mdi™). Samples were subjected to analyze with an amino acid analyzer (AminoTac™ JL500/V; JEOL). Intracellular amino acid concentrations were expressed as a percentage of dry cell weight.

### Fluorescent Microscopy

Wild-type yeast strains (BY4741) individually harboring pRS416-Bat1-yeGFP/pRS415, pRS416-Bat1-MTS-yeGFP/pRS415, pRS415-Bat2-yeGFP/pRS416, and pRS415-Bat2+MTS-yeGFP/pRS416 were cultured in SD+His/Met medium and middle and late log-phase cells were collected. Cell pellet was washed with distilled water and resuspended in distilled water containing 40 nM of MitroTracker® (Invitrogen) and incubated at 30°C for 30 min. Five µL of poly-l-lysine was coated on slides and dried up to promote the attachment of cells. Ten µL of cell suspension was applied on the slide, covered with coverslip and sealed with enamel coating. The localization of each protein was observed under a fluorescent microscope Axiovert 200M (Carl Zeiss). Images were captured with a HBO 100 Microscope Illuminating System (Carl Zeiss) digital camera.
